# Supplementary material for: Simultaneous Determination of Enantiomeric Purity and Organic Impurities of Dexketoprofen Using Reversed-Phase Liquid Chromatography—Enhancing Enantioselectivity through Hysteretic Behavior and Temperature-Dependent Enantiomer Elution Order Reversal on Polysaccharide Chiral Stationary Phases
Source: Int J Mol Sci. 2024 Feb 26;25(5):2697. doi: 10.3390/ijms25052697 (PMC10932108; doi:10.3390/ijms25052697)
Supplement: Supplementary file 1 [file ijms-25-02697-s001.zip › ijms-2877192-supplementary.pdf]

SUPPLEMENTARY MATERIALS

Simultaneous Determination of Enantiomeric and Organic Impurities of Dexketoprofen Using Reversed-Phase Liquid Chromatography. Fine-Tuning Enantioselectivity through Hysteretic Behavior and Temperature-Dependent Enantiomer Elution Order on Polysaccharide Chiral Stationary Phases

Máté Dobó[1], Gergely Dombi[1], István Köteles[1], Béla Fiser[2,3,4], Csenge Kis[5], Zoltán-István Szabó[5,6], Gergő

[1] Department of Pharmaceutical Chemistry, Semmelweis University, Budapest H-1085, Hungary;

[2] Institute of Chemistry, University of Miskolc, 3515 Miskolc-Egyetemváros, Hungary;

[3] Ferenc Rákóczi II. Transcarpathian Hungarian College of Higher Education, 90200 Beregszász, Transcarpathia, Ukraine

[4] Department of Physical Chemistry, Faculty of Chemistry, University of Lodz, Lodz, Poland

[5] Department of Pharmaceutical Industry and Management, George Emil Palade University of Medicine, Pharmacy, Science, and Technology of Targu Mures, Gh. Marinescu 38, Targu Mures, Romania

[6] Sz-imfidum Ltd, Lunga nr. 504, Romania

Correspondence: toth.gergo@semmelweis.hu

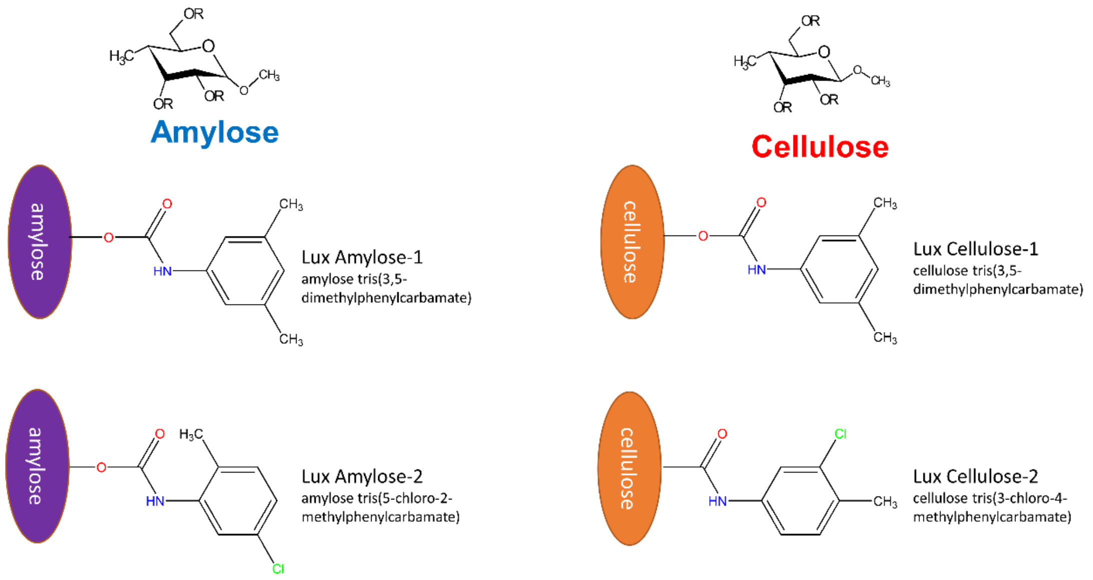

Supplementary Figure S1. The structure of the chiral selectors

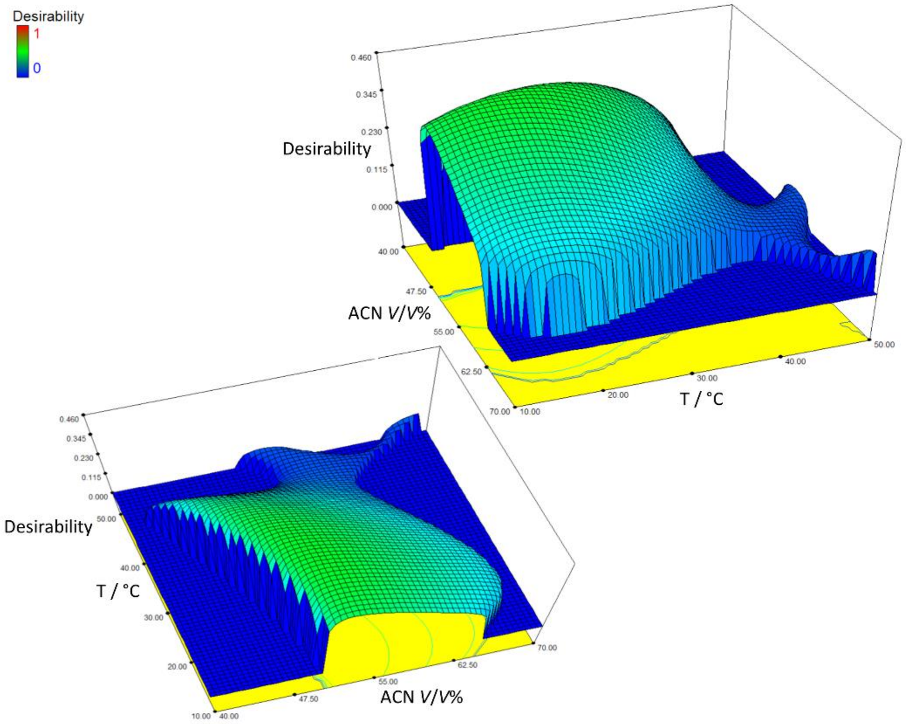

Supplementary Figure S2. Three-dimensional response surface plots obtained for desirability.

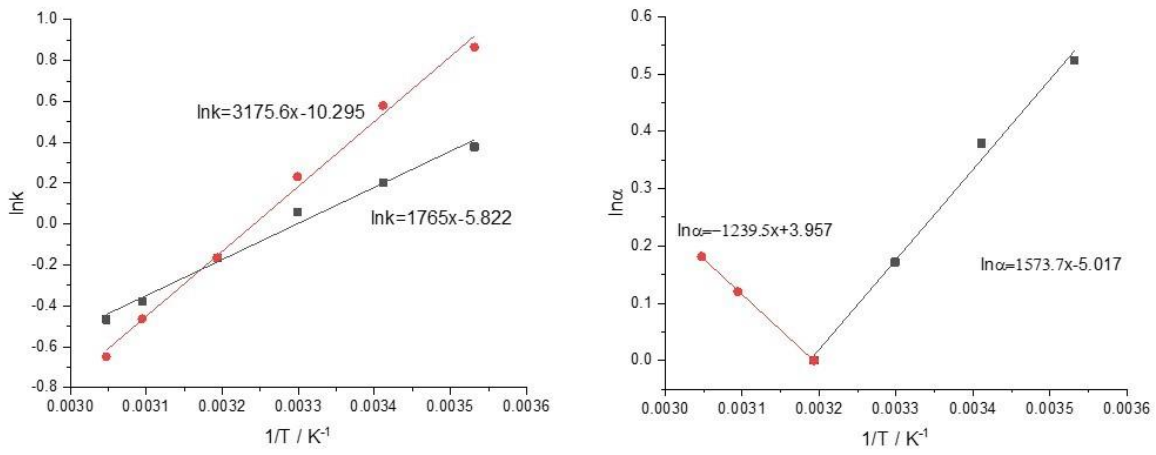

Supplementary Figure S3. lnk vs. 1/T (left) and lnα vs. 1/T (right) plots for the separation of ketoprofen enantiomers on Lux amylose-2 column. (Mobile phase: water/ACN/acetic acid 50/50/0.1 (v/v/v), flow rate: 1 mL/min)
